# Synthesis of Methylgenipin and Evaluation of Its Anti-Hepatic Injury Activity

**DOI:** 10.3390/molecules28124793

**Published:** 2023-06-15

**Authors:** Jingjing Wang, Yongwei Qiu, Yaohui Chen, Feng Zhou, Shuaikang Wang, Liping Chen, Yinfang Chen, Riyue Yu, Liping Huang

**Affiliations:** 1 School of Pharmacy, Jiangxi University of Chinese Medicine, Nanchang 330004, China; wangjingjing@jxutcm.edu.cn (J.W.); vvqiu517@163.com (Y.Q.); wsk2000127@163.com (S.W.); clp157707503482022@163.com (L.C.); chenyinfang_1984@126.com (Y.C.); yry59@126.com (R.Y.); 2 Jiangxi Provincial People’s Hospital, Nanchang 330012, China; yhchendoc@163.com (Y.C.); fancy-zhoufeng@163.com (F.Z.); 3 Jiangxi Provincial Key Laboratory of Pharmacology of Traditional Chinese Medicine, Nanchang 330004, China

**Keywords:** genipin, structural modification, methylgenipin (MG), ANIT, liver injury

## Abstract

Genipin has been the focus of research as a multifunctional compound for the treatment of pathogenic diseases. However, hepatotoxicity caused by oral genipin raises concerns about its safety. To obtain novel derivatives with low toxicity and efficacy, we synthesized methylgenipin (MG), a new compound, using structural modification, and investigated the safety of MG administration. The results showed that the LD_50_ of oral MG was higher than 1000 mg/kg, no mice died or were poisoned during the experiment in the treatment group, and there was no significant difference in biochemical parameters and liver pathological sections compared with the control. Importantly, MG (100 mg/kg/d) treatment for 7 days reduced alpha-naphthylisothiocyanate (ANIT)-induced increases in liver index, alanine aminotransferase (ALT), aspartate aminotransferase (AST), alkaline phosphatase (AKP), and total bilirubin (TBIL) levels. Histopathology demonstrated that MG could treat ANIT-induced cholestasis. In addition, using proteomics to investigate the molecular mechanism of MG in the treatment of a liver injury may be related to enhancing antioxidant function. Kit validation showed that ANIT induced an increase in malondialdehyde (MDA) and a decrease in superoxide dismutase (SOD) and glutathione (GSH) levels, while the MG pretreatments, both of which were significantly reversed to some extent, suggested that MG may alleviate ANIT-induced hepatotoxicity by enhancing endogenous antioxidant enzymes and inhibiting oxidative stress injury. In this study, we demonstrate that the treatment of mice with MG does not cause impaired liver function and provide an investigation of the efficacy of MG against ANIT-induced hepatotoxicity, laying the foundation for the safety evaluation and clinical application of MG.

## 1. Introduction

Liver disease is a cause of mortality and a heavy cost burden worldwide [1]. Acute liver failure (ALF) caused by foodborne poisons and infections led to the most serious threat in the past, while most modern liver diseases are caused by chronic pathogenic processes, such as alcoholic liver disease, nonalcoholic fatty liver disease (NAFLD), viral hepatitis, and drug-induced liver injury (DILI) [2]. The vast majority of regions in the Asian region are thought to encounter a severe burden of liver disease. It is estimated that 740,000 people are chronically infected with hepatitis B virus (HBV) in China compared to 170,000 in India [3]. Increasing experimental data have demonstrated that genipin has potential therapeutic effects on cholestasis, sepsis, acute liver injury caused by ischemia/reperfusion, and fulminant hepatitis, and its pharmacological properties include anti-inflammatory [4], antiviral [5], neuroprotective [6], hypoglycemic, lipid-regulating, and hepatoprotective cholagogue [7]. The hepatoprotective and cholagogue effect of genipin first came to the attention of scholars, whose research history dates back to the 1980s. Japanese team clarified that genipin enhances the distribution of Mrp2 protein to bile canaliculi and promotes mRNA and protein synthesis in hepatocytes, thereby enhancing bilirubin disposal in vivo [8,9]. In rats with α-naphthalene isothiocyanate-induced cholestasis, genipin ameliorated intrahepatic bile duct cholestasis and hepatocyte necrosis [10,11]. However, as early as about two decades ago, scientists such as Ozaki discovered the genitotoxic potential of genipin [12]. Later, it was found in experimental rats that genipin binds to some nucleophilic molecules at high doses, causing hepatotoxicity. Hepatotoxicity is characterized by elevated liver enzymes, liver weight, focal necrosis, and marked inflammatory infiltration of hepatocytes [13]. The main reason is that geniposide is hydrolyzed and converted to genipin under the action of intestinal flora, which is metabolized in the body, and the hemiacetal structure is unstable and ring-opened, which further binds to free glutathione, cysteine, and other amino acids in the body and destroys the relative homeostasis of protein levels, which, in turn, causes great toxicity to the liver [14,15].

The hepatotoxicity of genipin limits its clinical application, so we synthesized a new genipin derivative through structural modification, which can reduce the hepatotoxicity of genipin and exert hepatoprotective and cholagogue activities at the same time. Subsequently, we conducted a safety study and pharmacodynamic analysis of the new compound MG, focusing on the effect of MG on liver function, and we explored the mechanism of MG in the treatment of ANIT-induced liver injury in combination with proteomics techniques. This study helps to find the optimal effective dose of MG and promote its preventive and protective effects to translate into clinical applications, laying the foundation for the safety evaluation and clinical application of MG, so that MG is expected to be a potential drug instead of genipin in the treatment of liver injury.

## 2. Results

### 2.1. HPLC Idenification of MG

**Figure 1:**
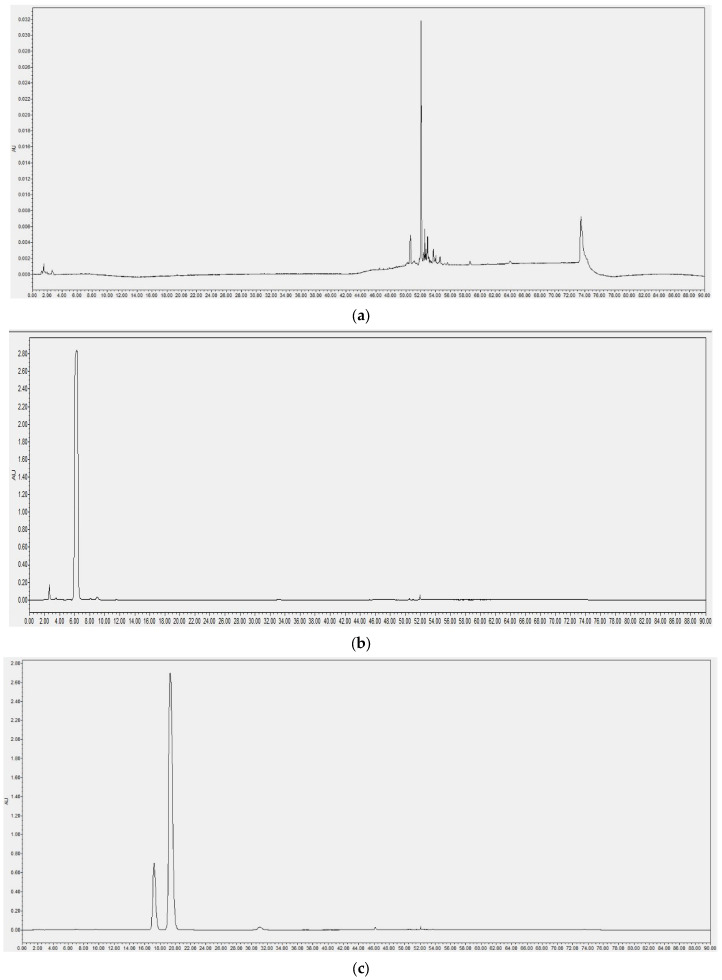
HPLC identification of MG. (**a**) Liquid chromatography of solvents. (**b**) Liquid chromatogram of Genipin. (**c**) Liquid chromatogram of MG.

**Figure 2:**
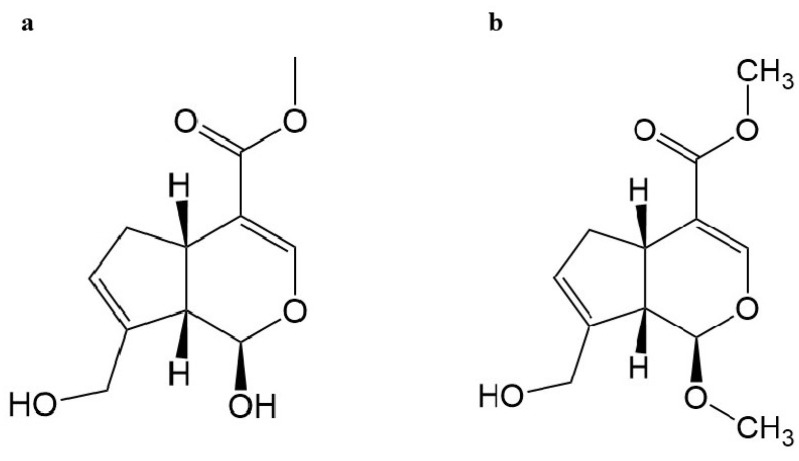
The structure of (**a**) genipin; and (**b**) methyl genipin (MG).

The results of the liquid phase detection of the solvent, genipin, and MG are shown in Figure 1a–c, and the results showed that the MG peak pattern was smooth under this chromatographic condition, the resolution was good, and there was no significant tailing. Impurities were monitored at the absorption wavelengths of genipin and MG, and we found that the impurity peaks differed greatly from the peak appearance time of the target product, and there was no impurity response at the retention times of these two compounds, which did not affect the results of their qualitative detection. The retention times were 6.3 min for genipin and 19.5 min for MG. It can be seen from the figure that two substances showed peaks in the peak appearance period of MG, and their peak area ratio was between 1:4 and 1:2. When the purity was verified by the liquid phase, these two substances were identified as a pair of isomers of MG based on the polar characteristics and peak area ratio of the compounds. Compared with the literature [16], it was determined that the component with a short retention time was S-MG, and the component with a long retention time was R-MG (Figure 2a,b). According to the peak area, the purity was calculated as 97.02% for genipin and 97.19% for MG products; among them, the S-MG content was 15.96% and the R-MG content was 81.23%.

### 2.2. Structure Determination of MG

After evaporating the purified product to dryness, 0.5 mL of deuterated chloroform (CDCl_3_) was used as a solvent to determine the hydrogen and carbon spectra of MG under nuclear magnetic resonance at 600 MHz. The ^1^H NMR signal of MG (Table 1) was analyzed in 90% yield, and R-MG C and H were assigned as follows: ^1^H NMR (600 MHz, chloroform-d) δ 7.51–7.50 (s, 1H), 5.84–5.81 (s, 1H), 4.49–4.44 (d, J = 8.1 Hz, 1H), 4.24–4.22 (s, 2H), 3.73–3.70 (d, J = 4.4 Hz, 3H), 3.59–3.56 (s, J = 1.0 Hz, 3H), 3.22–3.16 (m, 1H), 2.91–2.83 (m, 1H), 2.62–2.57 (m, 1H), and 2.11–2.03 (m, 1H).

**Figure 3:**
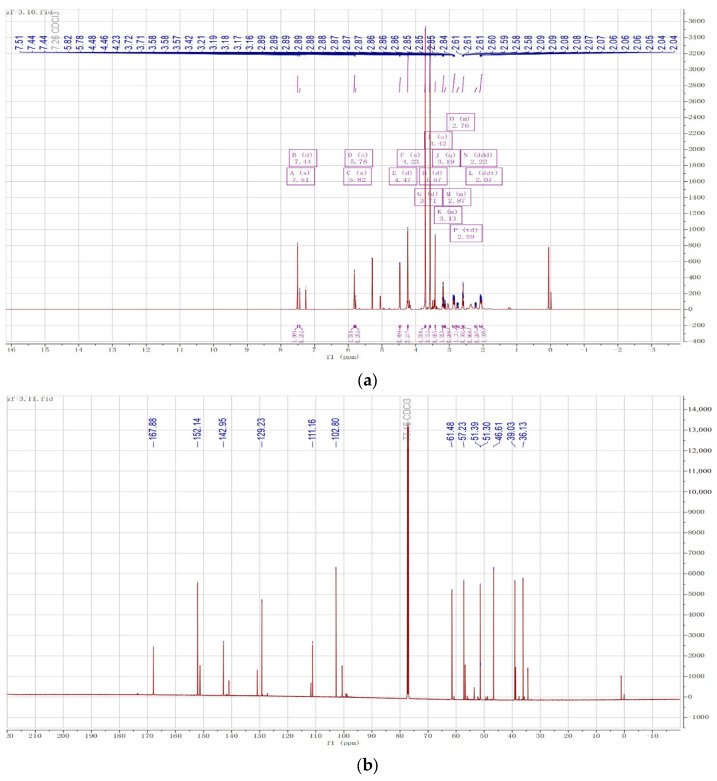
Structure determination of MG. (**a**) ^1^H-NMR of MG. (**b**) ^13^C-NMR of MG.

^13^C NMR (151 MHz, CDCl3) δ 36.1, 39.0, 46.6, 51.3, 57.2, 61.5, 102.8, 111.2, 129.2, 143.0, 152.1, and 167.9. The results of MG NMR are shown in Figure 3a,b.

### 2.3. Acute Hepatotoxicity of Methylgenipin in Normal Mice

**Figure 4:**
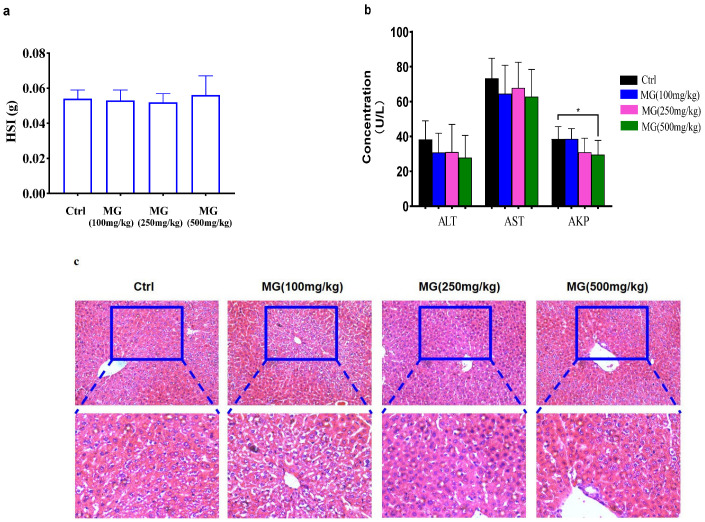
Evaluated acute hepatotoxicity of MG in normal mice. (**a**) Effect of MG on liver index in mice. (**b**) Biochemical parameters in serum after a single oral dose of MG, measured markers including ALT, AKP, AST, *n* = 10. (**c**) Representative HE images of livers from mice in different treatment groups, scale bar = 200 μm. Data shown are mean ± SD; compared to control, * *p* < 0.05, *n* = 10.

Oral doses of MG up to 1000 mg/kg body weight did not produce any toxicity-induced symptoms or mortality. The LD_50_ of MG in mice was greater than 1000 mg/kg body weight, while it has been previously reported in the literature that there is significant acute hepatotoxicity after oral administration of genipin ≥ 574 mg/kg in SD rats [17]. Thus, the modified MG is safer. Compared with the control group, there was no significant abnormality or death in the MG group. The results of the liver index showed that there was no significant difference in each MG treatment group compared with the control group, suggesting that high-dose MG did not increase the liver index in normal mice. No significant changes in serum albumin AKP and AST and ALT enzyme (Figure 4b) liver function parameters were observed after oral administration, and no damage was observed in HE staining (Figure 4c), suggesting that MG is not hepatotoxic in mice.

### 2.4. Assessment of Subacute Hepatotoxicity Test of MG in Normal Mice

**Figure 5:**
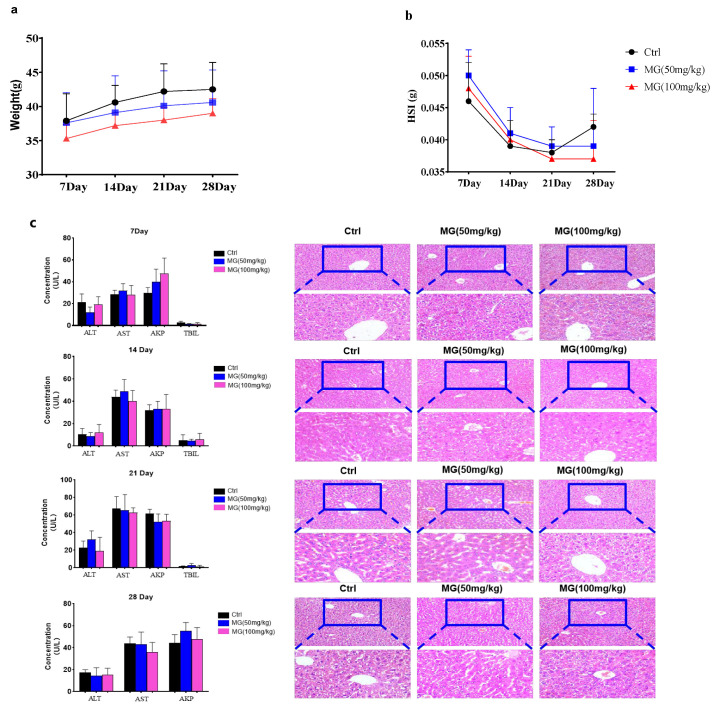
Assessment of subacute hepatotoxicity of MG in normal mice. (**a**) Trend diagram of MG on body weight of normal mice. (**b**) Trend diagram of liver coefficient of MG in normal mice. (**c**) Serum ALT, AST, AKP, TBIL levels in mice over 1–4 weeks and representative HE images of livers from mice in different treatment groups, scale bar = 200 μm, *n* = 10. Data shown are mean ± SD.

MG was administered continuously for 3 weeks followed by withdrawal, and no animal died during 1 week of observation. Mice in the control and treated groups showed an increasing trend in body weight, while the liver coefficient showed a decreasing trend with increasing time, and there were no significant differences between the groups (Figure 5a,b). The results of serum biochemical analysis showed (Figure 5c) that AKP was significantly decreased compared with the control group after administration on day 21, and no significant difference was observed in their ALT, AST, and TBIL plasma levels, indicating that MG did not affect the liver function parameters of normal mice. Histopathological findings (Figure 5c) showed that the cells in the control group were arranged tightly and neatly, the structure of liver tissue was normal, and there was no significant degeneration, necrosis, or inflammatory cell infiltration; with the extension of administration time, the cells in the MG-treated group were arranged neatly, and no significant degeneration or necrosis areas were observed. According to the above results, the pathological state of the liver in the MG-treated group was similar to that in the control group under long-term administration, indicating that MG did not induce liver injury in the liver of normal mice in vivo.

### 2.5. Therapeutic Effect of MG on ANIT-Induced Cholestatic Liver Injury

**Figure 6:**
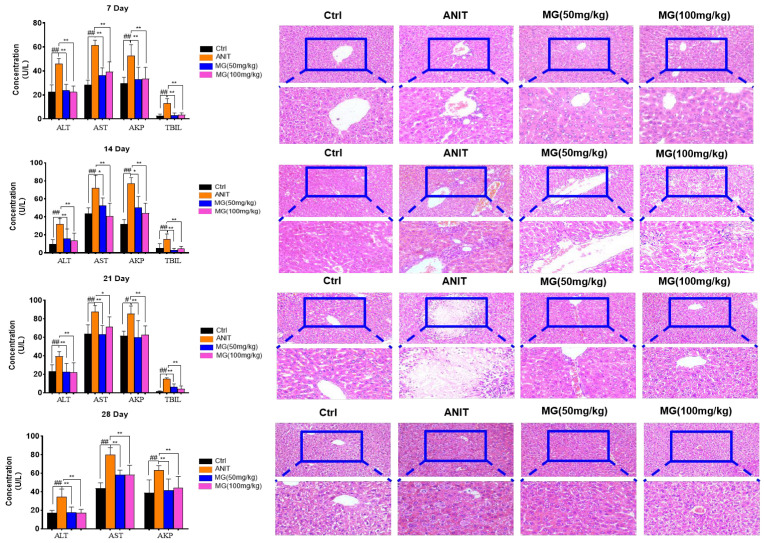
Therapeutic effect of MG on ANIT-induced cholestatic liver injury. ALT, AST, AKP, and TBIL levels in serum of mice over 1~4 weeks and representative HE images of livers of mice in different treatment groups, scale bar = 200 μm. Data are shown as mean ± SD; ## *p* < 0.05, # *p* < 0.01 versus control, ** *p* < 0.05, * *p* < 0.01 versus ANIT-treated, *n* = 10.

ANIT is metabolized by cytochrome P450 and undergoes GSH conjugation, which induces intrahepatic cholestasis, biliary epithelial cell necrosis, bile duct obstruction, and hepatocellular injury, features that mimic drug-induced liver injury in humans [18]. We first established a mouse model of acute cholestatic liver injury by treating mice with ANIT (70 mg/kg) for 12 h and administering them continuously for 7 days, 14 days, 21 days, and 28 days according to time points in order to observe the optimal administration time. During the administration period, the body weight of mice in the ANIT-induced model group showed a decreasing trend, and the MG-treated group dose-dependently recovered the body weight of mice (Table 2). Compared with the model group, the liver index of the MG-treated group was significantly decreased (Table 2), suggesting that a high dose of MG did not increase the liver index of normal mice but alleviated hepatomegaly, hyperplasia, congestion, and edema caused by ANIT in mice. At the end of this treatment period, we found that serum ALT, AST, AKP, and TBIL were significantly increased in experimental animals in the ANIT group compared with the control group (Figure 6a), indicating that ANIT causes more severe liver injury and leads to impaired uptake, binding, and excretion of bilirubin by hepatocytes. However, ANIT-induced increases in ALT, AST, TBIL, and AKP levels were effectively reversed by MG treatment (Figure 6a). In contrast, treatment with MG for 7 days effectively reduced ANIT-induced liver injury, and the therapeutic effect did not change significantly after increasing the days of administration. Histopathologically, the liver in the ANIT group was accompanied by massive periportal hemorrhage, diffuse vacuolation, inflammatory infiltration, and parenchymal necrosis (Figure 6a), and MG treatment was able to alleviate ANIT-induced liver injury in a dose-dependent manner. The above experimental results showed that MG could exert the best therapeutic effect on ANIT-induced acute liver injury after 7 days of administration.

### 2.6. Proteomic Study of MG in the Treatment of ANIT-Induced Liver Injury

#### 2.6.1. Screening for Differential Proteins

**Figure 7:**
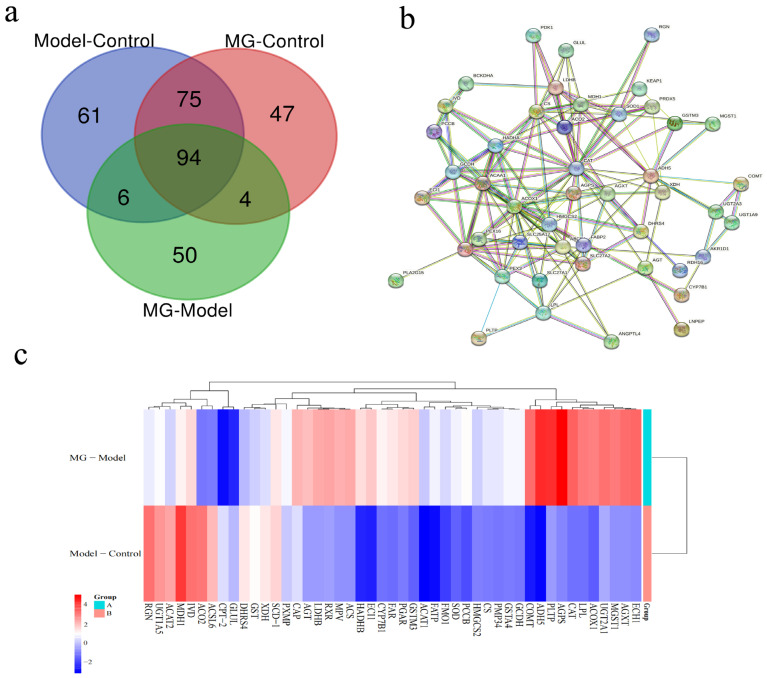
Screening of differential proteins. (**a**) Venn indicates differentially expressed proteins in cholestatic mice with or without MG treatment in the model versus control, MG versus control, and MG versus model groups. (**b**) Protein–protein interaction network analysis of differentially expressed proteins. (**c**) Heat maps showing trends in differentially expressed proteins in cholestatic mice with or without MG treatment. Associated Uniprot ID is provided on the right side of the heatmap. Red indicates up-regulation and blue indicates down-regulation.

In experiments with MG in ANIT-induced liver injury model mice, we found that MG exerted a better therapeutic effect after 7 days of treatment. However, the mechanisms involved in the protection of liver tissue by MG were not investigated. We then used proteomics to screen differential proteins in liver tissue and investigated the molecular mechanism of MG in the treatment of ANIT-induced liver injury from a proteomic point of view. RAW files of liver tissues collected by LC-MS from the 7-day control, model, and MG (100 mg/kg) treatment groups were imported into Protein Discovery software for proteomic identification. After a total of 2310 protein screening data were detected and quantified, 337 dynamically changing proteins were obtained in each different group (Figure 7a). The results of STRING database analysis showed that 51 differential proteins were included in the STRING database. After removing the isolated proteins (Figure 7b), the top ten proteins in the number of protein junctions were HMGCS2, SLC27A2, SOD1, HADHA, ADH5, CS, ACAA1, ACSL5, ACOX1, and CAT. Subsequently, the trend of differential protein expression in mice with liver injury treated with MG was further analyzed with a heat map (Figure 7c).

#### 2.6.2. Differential Protein GO and KEGG Analysis Results

**Figure 8:**
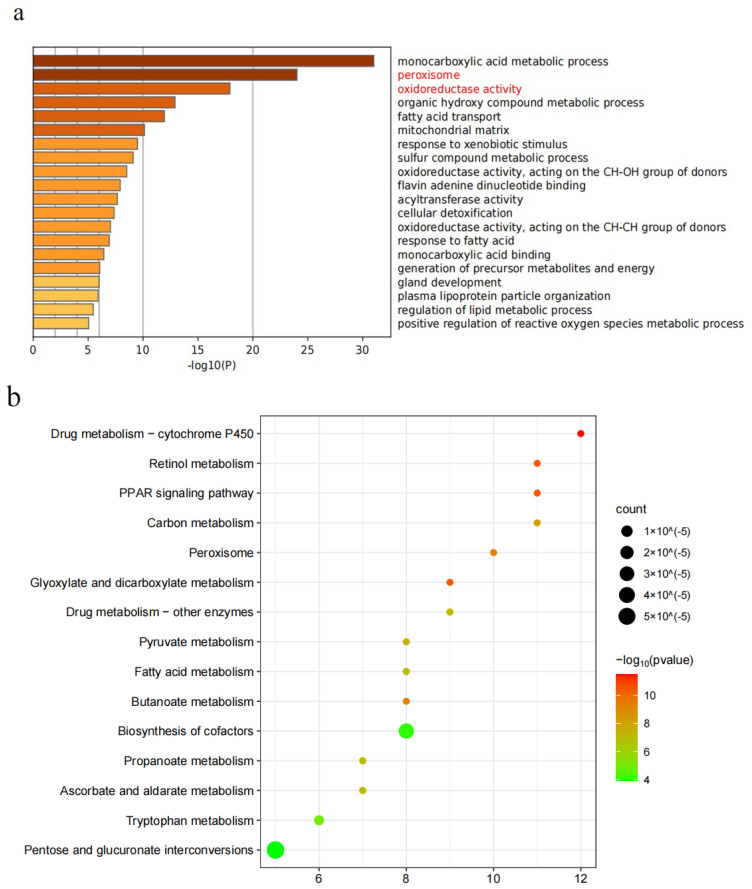
Differential protein GO and KEGG analysis results. (**a**) Cell composition, molecular function, and biological process analysis of differential proteins. (**b**) Enrichment analysis of database signaling pathways.

To further evaluate the function of these proteins, functional analysis of differential proteins was performed, and the classification results showed that molecular function and biological process analysis were related to monocarboxylic acid metabolic process, fatty acid metabolic process, peroxisome, oxidoreductase activity, fatty acid membrane process, fatty acid oxidation, and peroxisomal acid oxidation. In addition, multiple down-regulated proteins were associated with redox processes and lipid metabolism processes (Figure 8a). KEGG pathway analysis showed that the main pathways enriched in differentially expressed proteins were drug metabolism-cytochrome P450, glyoxylate, dicarboxylate metabolism, retinol metabolism, PPAR carbon signaling pathway, peroxisome, butanoate metabolism, ascruvate metabolism, ascorbate, alternate metabolism enzymes, and drug metabolism, the other top 10 most significant enrichment pathways (Figure 8b), indicating that MG may be involved in the treatment of α-naphthyl isothiocyanate-induced cholestasis mice through the above signaling pathways.

#### 2.6.3. Validation of Proteomic Critical Targets of MG against Liver Injury

**Figure 9:**
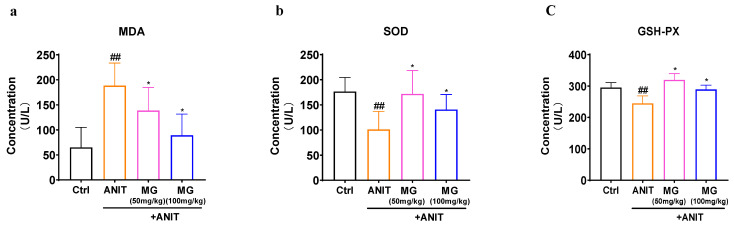
Validation of proteomic critical targets of MG against liver injury. Effect of MG on malondialdehyde (MDA), superoxide dismutase (SOD), and glutathione peroxidase (GSH-Px) levels in ANIT-induced hepatotoxicity in mice. (**a**) Liver tissue MDA level. (**b**) Liver tissue SOD level. (**c**) Liver tissue GSH-Px level. Data are shown as mean ± SD; ## *p* < 0.05, versus control, * *p* < 0.01 versus ANIT-treated, *n* = 10.

In proteomic studies, we found that ANIT affected bile acid synthesis and secretion and caused oxidative stress, while multiple proteins were involved in redox processes and down-regulated their related proteins in the signaling pathway of MG treatment liver injury. Therefore, we examined the effect of MG on antioxidant SOD, MDA, and GSH in the liver using kits. ANIT is metabolized by cytochrome P450 and undergoes GSH conjugation, thereby inducing intrahepatic cholestasis, biliary epithelial cell necrosis, bile duct obstruction, and hepatocellular injury. After liver injury, the expression level of MDA was significantly increased, while after MG treatment, the high expression of MDA was significantly decreased. In addition, GSH and SOD levels were decreased compared to controls in ANIT-induced injured livers, while they were effectively reversed in a dose-dependent manner following MG administration (Figure 9a–c).

## 3. Discussion

MG is a new synthetic compound modified by hydroxymethylation at position C-1 of genipin and can be regarded as a new component to replace genipin in exerting hepatoprotective efficacy. Toxicological testing in animal studies is frequently used to assess the possible negative effects of novel compounds on humans [19], and the semi-lethal dose LD_50_ refers to the dose that can cause death in 50% of experimental animals. In acute hepatotoxicity studies, no adverse effects or mortality were observed in mice after a single oral dose of MG, and it can be concluded that the oral dose of MG in mice is LD_50_ > 1000 mg/kg, while there was significant acute hepatotoxicity after oral administration of genipin ≥ 574 mg/kg in SD rats [17]. Therefore, MG is much greater than genipin oral safety. In addition, we found no significant differences in body weight, liver index, biochemical parameters, and liver sections between mice treated continuously for 28 days and controls. The above experimental results showed that MG did not produce any acute or subacute toxicity at a dose of 1000 mg/kg, and MG may become a new safe compound for the treatment of liver damage. To assess MG in more depth and potentially guide its future drug application, the pharmacological effects of MG were analyzed in this study. The liver is one of the most important organs in the human body and can promote the biotransformation of drugs [20]. ALT, AST, AKP, and TBIL are serum biomarkers of normal function and specific markers of hepatocyte degeneration [21,22], and the increase in serum ALT and AST marks liver injury and reflects the occurrence of hepatotoxicity [19]. The pharmacological experiment of liver protection was divided into four time points, and the optimal administration time of MG was investigated through continuous gavage for 7, 14, 21, and 28 days. The experimental results showed that ANIT (70 mg/kg) increased the levels of serum ALT, AST, TBiL, and AKP, and MG treatment attenuated the serum levels in a dose-dependent manner, which was further confirmed using histopathological analysis results. Administration for 7–28 days can prevent acute liver injury induced by α-naphthalene isothiocyanate (ANIT) in extrahepatic cholestasis, and considering the patient’s compliance, we recommend taking the drug continuously for 7 days. We then performed proteomic analysis of liver tissue on day 7 of treatment to analyze the mechanism of action of MG in the treatment of liver injury at the molecular level.

The molecular mechanism of ANIT-induced intrahepatic cholestasis is cytochrome P450 metabolism and GSH conjugation, and long-term cholestasis can lead to hepatobiliary obstruction or even liver failure [23]. Recently, increasing research has reported that the pathogenesis of intrahepatic cholestasis is closely related to oxidative stress and abnormal fatty acid metabolism [24,25,26,27]. Previously, genipin has been shown to treat liver fibrosis by ameliorating oxidative stress injury [28]. After consulting the relevant literature, the research group used Nano-LC-LTO-Orbitrap to explore and analyze the differential protein expression profile of MG in the treatment of liver injury and verified the key protein expression using a kit according to the omics results. A total of 94 significantly differentially expressed proteins were identified, and critical protein expression was detected using kits based on omics results. KEGG pathway analysis showed that MG was involved in Peroxisome, PPAR signaling pathway and fatty acid degradation, metabolism, and biosynthesis. The PPAR signaling pathway and fatty acid degradation have been reported to be involved in several metabolic modalities, such as pyruvate metabolism, carbon metabolism, and retinol metabolism. Among them, cytochrome P450 is closely related to drug metabolism in carbon metabolism. Excessive secretion of bile acids induces oxidative stress to disrupt the internal normal oxidative state and stimulate mitochondria to produce reactive oxygen species (ROS), and the toxicity of excessive reactive oxygen species production can lead to hepatocyte death [26]. It has been shown that genipin can act as an antioxidant to promote HO-1 protein increase and inhibit NO release in the liver. Thus, it is speculated that synthetic MG similarly reduces the imbalance between oxidative and antioxidant systems [24]. In addition, GSH-Px was involved in oxidative defense [29], and the kit results showed that MG could increase the expression level of GSH-Px in liver tissue. The above results showed that the mechanism of MG in treating liver injury may be related to alleviating oxidative stress injury and reducing ROS levels to enhance antioxidant function.

In this study, we discovered that structurally modified genipin was non-toxic or minimally toxic to the liver, providing valuable information on acute and subacute oral hepatotoxicity in MG. More importantly, MG has pharmacological effects and can alleviate liver injury caused by ANIT-induced acute intrahepatic cholestasis, which may be related to the enhancement of antioxidant function and regulation of abnormal fatty acid metabolism by MG, and MG is expected to be a potential key compound for the prevention and treatment of cholestatic liver injury.

## 4. Materials and Methods

### 4.1. Reagents and Chemicals

Genipin was purchased from Xian Green Biological Co., Ltd. (Xi’an, China, batch number 190604, purity 98%), and methanol and acetonitrile were obtained from Merck KGaA (Hongkong, China, Chromatographic grade, purity ≥99.9%, batch number I0997130907, I1098407025). Concentrated hydrochloric acid, petroleum ether, ethyl acetate, dichloromethane, formaldehyde, and dimethyl benzene were purchased from Xi long Scientific Co., Ltd. (Guangzhou, China, batch number 1803231, 1907041, 20180801, 1903302). Fumed silica (Specification: 100–200, 200–300) was obtained from Qingdao Ocean Chemical Factory (Shandong, China), and sodium bicarbonate was purchased from Compuware Technology Co., Ltd. (Beijing, China, batch number 20120802). Chloride standard and magnesium sulfate were purchased from Yong da Chemical Reagent Development Centre (Tianjin, China, 20090223, 20090704). Chloroform-d was purchased from Cambridge Isotope Laboratories. Inc (Boston, USA, batch number PR3065/01259CL1). Pentobarbital sodium salt was obtained from Merck KGaA (Hongkong, China) CMC-Na, and PMSF was purchased from Solarbio (Beijing, China, batch number No.1224M022). ANIT and trifluoroacetic acid were purchased from Macklin Maclean Biotech Co., Ltd. (Shanghai, China, batch number c11230778). Food-grade olive oil was purchased from Yihai Grain and Oil Industry Co., Ltd. (Guangzhou, China, batch number 20190807). ALT, AST, AKP, TBIL, SOD, MDA, GSH-PX, and HE staining solution were purchased from Nanjing Jingzhu Bio-technology Co., Ltd. (Nanjing, China, batch number 20200906, 20200907, 20200907, 20201209, 20201212, 20201218, 20201218, 20200824). Ethanol was purchased from Hengxing Chemical Reagent Co., Ltd. (Tianjin, China, batch number 20190215). Acetone was purchased from Sinopharm Chemical Reagent Co., Ltd. (Shanghai, China, Analytically pure, batch number 20141008). RIPA High-Efficiency Lysis Solution and BCA Protein Assay kit were purchased from Kangwei Century Biotechnology Co. (Nanjing, China, batch number 40537). DTT was purchased from Thermo Fisher Scientific (Waltham, MA, USA, batch number QE217272A). IAA, ammonium bicarbonate, acetic acid, and formic Acid were purchased from Sigma Aldrich (St. Louis, MO, USA, batch number SLCD4031). Pancreatic enzymes were obtained from Madison, WI, USA, batch number 0000436483. Ammonium bicarbonate, acetic acid, and formic acid were obtained from Sigma-aldrich (Shanghai, China, Chromatographic grade, batch number BCBN6056V, BCBN4472V, BCBP4740V).

### 4.2. Synthesis of MG

Reaction principle: Genipin is a hemiacetal structure compound, the -OH of hemiacetal is unstable, and it is easy to dehydrate and condense with another molecule of alcohol to form acetal. Based on this principle, genipin was acetal reacted with methanol under acidic conditions to control the reaction conditions, and MG was synthesized [30,31,32]. Reaction process: Genipin and methanol were reacted in a ratio of 1:16 (g/g) in the following order: genipin (2g)methanol (40 mL)concentrated hydrochloric acid (8 drops), added into a 500 mL round-bottom flask, the oil bath was heated to 60 °C, during which stirring was continued, the condenser was connected to reflux, and the reaction was performed for 3 h. The reaction progress was monitored using a thin-layer spot plate. After the reaction was completed, the methanol was evaporated to dryness using a rotary evaporator. Then, 20 mL of dichloromethane (CH_2_Cl_2_) was added, transferred to an EP tube, and clear saturated bicarbonate (NaHCO_3_) aqueous solution was added. The mixture was extracted, allowed to stand for layering, and the lower CH_2_Cl_2_ phase was retained. Next, 10 mL of CH_2_Cl_2_ was added to the NaHCO_3_ aqueous phase, shaken to deflate, allowed to stand for layering, and the lower CH_2_Cl_2_ phase was retained again. This above process was repeated three times. Afterwards, 10 mL of saturated NaCl aqueous solution was added to the combined CH_2_Cl_2_ phase that was retained. The mixture was extracted three times, and the lower CH_2_Cl_2_ phase was retained. Finally, anhydrous magnesium sulfate was added, and after 2 h, the mixture was filtered. The solvent was evaporated at 60 °C to obtain the initial product. The product was then analyzed for initial purity using liquid phase chromatography.

### 4.3. Purification of MG

#### 4.3.1. Column Loading

Because there is little difference in molecular weight and polarity between genipin and MG, the silica gel column chromatography separation method was selected for separation and purification. Column size was chosen based on loading volume. In order to ensure the separation effect of the silica gel column, a wet packing column was used in this experiment. The steps are as follows: First, add silica gel to excess petroleum ether to make a dilute suspension and slowly add along the inner wall of a chromatographic column; try not to produce bubbles; after silica gel is slowly deposited according to gravity, add suspension again until the height of silica gel after deposition is 2/3 of the total height of the chromatographic column. At this point, the number of theoretical plates is >3000.

#### 4.3.2. Loading

The synthesized initial product was dissolved in a small amount of CH_2_Cl_2_ and slowly added along the inner wall of the column; the valve under the column was closed, and the mobile phase was added after the product CH_2_Cl_2_ solution was allowed to wet the silica gel column for a period of time. The mobile phase was a petroleum ether-ethyl acetate system. The elution gradient was 5:1–4:1–3:1–2:1–9:5–8:5–7:5–6:5–1:1. The elution was monitored using thin-layer chromatography.

#### 4.3.3. Qualitative NMR

AVANCE III HD 600 MHz NMR spectrometer was purchased from Bruker, Switzerland. The purified product was dissolved in 0.5 mL deuterated chloroform (CDCl_3_) and loaded into a nuclear magnetic resonance tube after evaporating the solvent and sent to the Large Instrument and Equipment Sharing Center of Jiangxi University of Chinese Medicine for nuclear magnetic resonance hydrogen spectroscopy and nuclear magnetic resonance carbon spectroscopy.

#### 4.3.4. HPLC Quantitation

Accurately weigh 9 mg genipin and dissolve in 1.5 mL acetonitrile; prepare 1.5 mL of 6.0 mg/mL sample. Add to the liquid vial after passing through the 2 μm filter membrane. After evaporating the synthesized and purified product to dry the solvent, 10 mg was accurately weighed and dissolved in 2 mL acetonitrile to prepare a 5 mg/mL sample, which was passed through a 2 μm filter membrane and added to a liquid vial. The pure solvent acetonitrile was passed through a 2 μm membrane and added to the liquid vial. Then, 2695–2998 analytical high-performance liquid chromatograph (American Water, Milford, MA, USA) separation C_18_ reverse-phase column (4.6 × 250 mm, 5 μm, Agilent, Santa Clara, CA, USA) was used. Mobile phase: acetonitrile (A)-water (B); gradient elution: 0~90 min, 20% A, injection volume 10 μL; detection wavelength: 238 nm [33,34], total running time: 0~90 min; column temperature: 30 °C.

### 4.4. Animals

**Figure 10:**
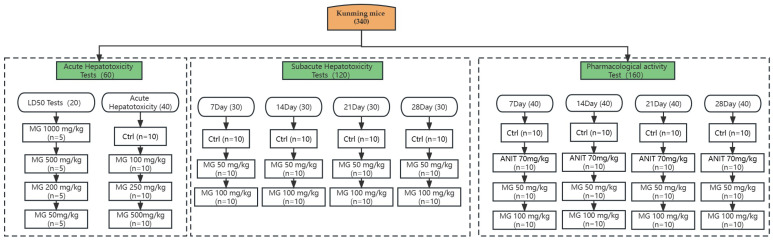
Overall animal group design diagram.

An acute toxicity test was performed in 60 SPF normal Kunming mice weighing 20 ± 2 g, half males and half females, and a subacute toxicity test was performed in 120 SPF normal Kunming mice weighing 20 ± 2 g, half males and half females, for 7 days, 14 days, 21 days, and 28 days. Pharmacological activity tests were performed on 160 SPF-healthy Kunming mice weighing 20 ± 2 g, half male and half female. The overall animal group design is shown in Figure 10 below. All animals were purchased from the Laboratory Animal Center of Jiangxi University of Traditional Chinese Medicine. Animal production and use license number: SCXK (Gan) 2018-0003; animal quality certificate number: 0002173. Bedding and feed were provided by the Laboratory Animal Center of Jiangxi University of Traditional Chinese Medicine. I and members of the project team strictly abided by the internationally accepted animal welfare and ethical standards, implemented the laws, regulations, and policies of the State and Jiangxi Province on laboratory animal management, and treated laboratory animals well. Jiangxi University of Chinese Medicine‘s ethics committee approved the study (JZLLSC 2019-391), and the Animal Ethics Committee approved 340 animals. All animals were maintained on a 12 h light/12 h dark cycle at 25 ± 2 C and 60 ± 20% relative humidity with unrestricted access to a standard laboratory diet and water except during fasting periods. Animals were acclimated to this environment for 1 week before dosing.

#### 4.4.1. Acute Hepatotoxicity Tests

Twenty SPF-healthy Kunming mice were randomly divided into 4 groups, with 5 mice in each group. MG solutions at 1000 mg/kg, 500 mg/kg, 200 mg/kg, and 50 mg/kg were prepared and administered intragastrically to mice, and the mortality of animals in each group was recorded for LD_50_ calculation. In the formal experiment, 40 SPF-healthy Kunming mice were randomly divided into 4 groups, half male and half female, and randomly assigned according to the body weight of the mice. The group design was: control; 10 animals in each group of MG (100 mg/kg/d), MG (250 mg/kg/d), and MG (500 mg/kg/d). MG was dissolved in 0.5% sodium carboxymethylcellulose solution at doses of 100 mg/kg, 250 mg/kg, and 500 mg/kg, respectively. After fasting for 12 h, the animals in each group were intragastrically administered with the corresponding dose of the drug, and the control was administered with saline. Within 24 h after administration, the activity, coat color, diet, excretion, and death of experimental animals in each group were observed and recorded. After the last administration, the animals fasted for 12 h. Following anesthesia of mice with 0.5% pentobarbital sodium salt (IP), blood samples were collected from eyeballs and centrifuged at 3500 rpm for 15 min to obtain serum for biochemical indicators. Liver organs were excised and weighed and fixed in 10% neutral formaldehyde for histopathological diagnosis.

#### 4.4.2. Subacute Hepatotoxicity Test

One hundred and twenty SPF-healthy Kunming mice were randomly divided into 4 groups according to body weight, with thirty mice in each group. Intragastric administration was performed for 7 days, 14 days, 21 days, and 28 days according to time points, of which day 28 was designed as the total time for 7 days of recovery after 21 days of administration. Animals at each time point were randomly divided into 3 groups according to body weight, with 10 animals in each group. That is, control; MG (50 mg/kg/d); MG (100 mg/k/d); MG was dissolved in 0.5% sodium carboxymethylcellulose solution at a concentration of 50 mg/mL. The doses administered were 50 mg/kg and 100 mg/kg. Animals in the treatment group were intragastrically administered with the corresponding dose of the drug, and animals in the control group were intragastrically administered with saline. During the administration period, the death and body-weight change of animals in each group over the administration time were recorded. After the last administration, the animals fasted for 12 h. 

#### 4.4.3. Therapeutic Effect of MG on ANIT-Induced Cholestatic Liver Injury

One hundred and sixty mice (half male and half female, ten mice in each group) were intragastrically administered for 7 days, 14 days, 21 days, and 28 days according to the time points, and each time point was randomly divided into 4 groups according to the animal body weight, control, ANIT (70 mg/kg) group, ANIT + MG (50 mg/kg/d), and ANIT + MG (100 mg/kg/d), which were used to investigate the duration of MG administration for the treatment of the liver injury. MG was dissolved in 0.5% sodium carboxymethylcellulose solution at a concentration of 50 mg/mL. The doses administered were 50 mg/kg and 100 mg/kg. Animals in each dose group were intragastrically administered with the corresponding dose of the drug, and animals in the control group were intragastrically administered with saline. The model animals were intragastrically administered with ANIT (70 mg/kg) to establish acute jaundice models according to the results of preliminary experiments, and 12 h after modeling, the animals in each treatment group were intragastrically administered with the corresponding dose of the drug, and the animals in the model group were intragastrically administered with saline. Repeated ANIT interventions were required every 3 days to reinforce the model. During the administration period, the death and body-weight change of animals in each group over the administration time were recorded. After the last administration, the animals fasted for 12 h. Following anesthesia of mice with 0.5% pentobarbital sodium salt (IP), blood samples were collected from eyeballs and centrifuged at 3500 rpm for 15 min to obtain serum for biochemical indicators. Liver organs were excised and weighed, fixed in 10% neutral formaldehyde for histopathological diagnosis, and stored in a −80 °C freezer for proteomic studies.

### 4.5. Determination of LD_50_ in Normal Mice

Genipin LD_50_ and its 95% confidence interval were calculated according to the modified Kirschner method, and the calculation formula was: log LD_50_ = X_m_ −(+) $\;\frac{1}{2}\sum_{j}^{i}\;$(p_j_ + p_j + 1_).

Hepatic Index Calculation

Hepatic index = liver weight/body weight × 100%(1)

### 4.6. Biochemical Indicators

Serum biochemical parameters of alanine aminotransferase (ALT), aspartate aminotransferase (AST), alkaline phosphatase (AKP), total bilirubin (TBIL), superoxide dismutase (SOD), malondialdehyde (MDA), and glutathione peroxidase (GSH-PX) were measured according to the kit instructions. 

### 4.7. Hepatic Pathology Experiments

A histopathological examination of liver tissues was performed. Tissues fixed in 10% formalin were immersed in paraffin blocks, cut into 5 mm thin sections, mounted on glass slides, and stained with hematoxylin and eosin (HE). Tissue sections on slides were observed and photographed using a light microscope (DMI3000B Inverted Fluorescence Microscope: Leica, Germany).

### 4.8. Liver Tissue Protein Extraction and Lysis

Three liver tissue samples were randomly selected from each group and quickly removed from the −80 °C freezer, and 0.1 g was weighed. Place the tissue into the rinse solution, suck the water on the tissue surface, and then place it into a 2 mL EP tube; place the steel bead, and add 1 mL lysis solution. All operations are required to be performed at 4 °C. Thus, 1200× *g* was ground for 30 s and repeated twice. The EP tubes were placed in an ice box, shaken on a shaker for 40 min, placed in a high-speed centrifuge, centrifuged at 14,000× *g* for 20 min at 4 °C, and the supernatants were taken, dispensed, and stored at −20 °C until use.

### 4.9. Protein Quantitation

According to the operating instructions of the BCA protein quantitative kit, prepare the protein standards and working solutions at the corresponding concentrations, add them into a 96-well plate, detect the absorbance at a wavelength of 570 microplate reader after sufficient reaction, and draw the standard curve. The protein supernatant was diluted 100-fold, and the absorbance of the diluted samples was measured according to the kit operating instructions, with 3 wells in parallel for each sample. Substitute the measured results into the standard curve and calculate the sample mother solution protein concentration.

### 4.10. Gene Ontology, Pathway Analysis, and Interactions between Proteins

Liver tissue proteins were detected using a Nano ESI-LC-MS combined system, and eggs were identified and qualitatively analyzed for relative quantification of proteins using Max Quant software. Gene ontology analysis was performed on the list of selected differential proteins using the Metascape database https://metascape.org (accessed on 3 March 2023). Pathway analysis used the DAVID database https://david.ncifcrf.gov/ (accessed on 3 March 2023). Network analysis was performed to assess protein–protein interactions using STRING database version 11.5 https://cn.string-db.org/ (accessed on 8 March 2023).

### 4.11. Statistical Methods

The data were presented as mean ± SD. GraphPad Prism 8.0.2 software (GraphPad Software, USA) was used to analyze the data. One-way analysis of variance (ANOVA) was used for statistical analysis, followed by Tukey’s multiple comparison test. * indicates comparison with control group, and # indicates comparison with model group, in which ** *p* < 0.05, * *p* < 0.01, ## *p* < 0.05, ## *p* < 0.01. *p* < 0.05 was considered statistically significant.

## 5. Conclusions

In this study, we found that genipin after structural modification into MG has higher oral safety, and histopathological, proteomic, and biochemical methods were used to find that its hepatoprotective effect was significant and it did not damage the liver of normal mice; its potential mechanism of action may be through MG-mediated lipid metabolism and oxidative stress. The above results suggest that MG may become an alternative to genipin as a candidate for the treatment of liver injury. However, additional experiments and further clinical studies are needed for safe therapeutic use.

## Figures and Tables

**Table 1:** ^1^H-NMR and ^13^C-NMR signal of MG.

Position	δH	δC
1	3.70 (d, J = 4.4 Hz, 3H)	57.2
2	4.44 (d, J = 7.8 Hz, 1H)	102.8
3	7.50 (s, 1H)	152.1
4	—	111.2
5	—	167.9
6	3.56 (s, J = 1.0 Hz, 3H)	51.3
7	2.58 (m, 1H)	36.1
8	2.05 (m, 1H), 2.84 (m, 1H)	39.0
9	5.81 (s, 1H)	129.2
10	—	143.0
11	3.16 (m, 1H)	46.6
12	4.22 (s, 2H)	61.5

**Table 2:** Effects of MG on body weight and liver index (liver weight/body weight %).

Groups	7Day		14Day		21Day		28Day	
	Weight (g)	Liver Index (%)	Weight (g)	Liver Index (%)	Weight (g)	Liver Index (%)	Weight (g)	Liver Index (%)
Ctrl	30.4 ± 3.97	0.028 ± 0.006	34.3 ± 2.68	0.033 ± 0.004	34.5 ± 3.45	0.033 ± 0.004	36.0 ± 4.67	0.037 ± 0.009
ANIT	33.8 ± 2.49	0.032 ± 0.004 ^##^	33.5 ± 1.47	0.043 ± 0.002 ^##^	34.2 ± 3.23	0.046 ± 0.006^##^	37.7 ± 4.45	0.048 ± 0.004 ^##^
MG (50mg/kg)	31.2 ± 4.05	0.029 ± 0.002 **	35.3 ± 4.19	0.039 ± 0.003 *	36.5 ± 3.72	0.037 ± 0.003 **	39.5 ± 4.65	0.037 ± 0.003 **
MG (100mg/kg)	31.9 ± 3.97	0.029 ± 0.002 *	35.2 ± 2.96	0.040 ± 0.003 *	36.9 ± 4.19	0.040 ± 0.005 *	38.2 ± 5.97	0.041 ± 0.004 *

^##^*p* < 0.01, compared to the control group; * *p* < 0.05, ** *p* < 0.01, compared to the ANIT group.

## Data Availability

Data is contained within the article.

## Sample Availability

Samples of the compounds are not available from the authors.
